# Potential Anticarcinogenic Effects From Plasma of Older Adults After Exercise Training: An Exploratory Study

**DOI:** 10.3389/fphys.2022.855133

**Published:** 2022-07-06

**Authors:** Alessandra Peres, Gisele Branchini, Bruna Marmett, Fernanda Bordignon Nunes, Pedro R.T. Romão, Tiago Olean-Oliveira, Luciele Minuzzi, Mateus Cavalcante, Viviane Elsner, Fabio Santos Lira, Gilson Pires Dorneles

**Affiliations:** ^1^ Laboratório de Imunologia Celular e Molecular, Departamento de Ciências Básicas da Saúde, Universidade Federal de Ciências da Saúde de Porto Alegre, Porto Alegre, Brazil; ^2^ Programa de Pós-graduação em Patologia, Universidade Federal de Ciências da Saúde de Porto Alegre—UFCSPA, Porto Alegre, Brazil; ^3^ Exercise and Immunometabolism Research Group, Postgraduation Program in Movement Sciences, Department of Physical Education, Universidade Estadual Paulista (UNESP), Presidente Prudente, Brazil; ^4^ Faculty of Sports Science and Physical Education, Research Center for Sports and Physical Activity, University of Coimbra, Coimbra, Portugal; ^5^ Programa de Pós-graduação em Fisiologia, Universidade Federal do Rio Grande do Sul, Porto Alegre, Brazil

**Keywords:** prostate cancer, immune response, aging, exercise training, inflammation, mitochondria

## Abstract

**Aim:** To evaluate the impact of exercise training plasma on *in vitro* prostate cancer cell viability and proliferation.

**Methods:** PC3 prostate cancer cells were incubated with plasma obtained from young men with high and low physical fitness (PF) (high PF, *n* = 5; low PF, *n* = 5) and with the plasma collected from institutionalized older adults (*n* = 8) before and after multimodal exercise training. Cell viability and proliferation, mitochondria membrane polarization, reactive oxygen species (ROS) generation, and apoptosis were evaluated after the cell treatment with plasma. Systemic cytokines were evaluated in the plasma of institutionalized older adults submitted to an exercise training protocol.

**Results:** Plasma from high-PF men lowers both cell viability and proliferation after the incubation time. PC3 cells also presented lower cell viability and diminished rates of cell proliferation after the incubation with post-training plasma samples of the older adults. The incubation of PC3 cells with post-training plasma of older adults depolarized the mitochondrial membrane potential and increased mitochondrial reactive oxygen species production. Post-training plasma did not change apoptosis or necrosis rates in the PC3 cell line. Multimodal exercise training increased the plasma levels of IL-2, IL-10, IFN-α, and FGF-1 and decreased TNF-α concentrations in institutionalized older adults.

**Conclusion:** Adaptations in blood factors of institutionalized older adults may alter cell viability and proliferation by targeting mitochondrial ROS in a prostate cancer cell line.

## Introduction

Biological aging impacts several aspects of the host immune system, characterized by a chronic inflammatory state associated with an accumulation of senescent exhausted myeloid and lymphoid cells (Bauer and De la Fuente, 2013). The chronic increases in inflammatory molecules are associated with the etiology and clinical course of most age-related diseases and mortality, including several cancer types (Barbé-Tuana et al., 2020). In this sense, prostate cancer becomes more prevalent in aging men, once the enhancement in the innate and adaptive inflammatory response contributes to prostate carcinogenesis (Taverna et al., 2014).

Emerging data indicate that physical exercise may have positive effects on the prevention and treatment of prostate cancer (Kim et al., 2021). Both preclinical and clinical studies described several immunological and physiological exercise-mediated adaptations that may prevent or attenuate prostate cancer progression, including enhanced T-cell and natural killer functional activity as well as the modification of systemic biochemical molecules (Kim et al., 2021). Furthermore, changes in systemic inflammation and the composition of blood factors induced by exercise training adaptations may directly impact cancer cell viability. Past data highlighted the role of acute exercise-conditioned serum or plasma from young and aged humans in the decrease of cancer cell viability, indicating that changes in blood molecules (i.e., hormones, cytokines, and reactive oxygen species) contribute to the anticarcinogenic potential during acute bouts (Hwang et al., 2020; Soares et al., 2021; Orange et al., 2022).

Decreases in prostate cancer cell viability induced by pharmacological drugs are mediated by changes in mitochondrial functions (Seo et al., 2019). In fact, mitochondria are emerging players in the tumorigenic process by maintaining the energetic capabilities of cancer cells (Boland, Chourasia, and Macleod, 2013). In prostate cancer, it is now clear that mitochondria are involved in the malignant process, cell proliferation aggression, and metastasis formation (Zichri et al., 2021). Mitochondria dysfunction and reactive oxygen species (ROS) generation are important events to reduce cancer cell viability (Wu et al., 2011). Modifications in mitochondrial activity lead to the expression of the cell cycle inhibitor p53, cell cycle arrest at the G2/M phase, DNA fragmentation, and subsequent cell death induction (Wu et al., 2011). Furthermore, *in vivo* observations demonstrated that ROS could trigger tumor apoptosis through increasing lipid, protein, and DNA damage within the tumor (Xie et al., 1995). It is interesting to note that chemotherapeutic agents induce ROS-induced lipid peroxidation and apoptosis in tumor cells (Jana et al., 2014). Exercise changes the redox state in several tissues and may be an important non-pharmacological agent to contribute to the tumor cell's growth, acting as a preventive agent or as a rehabilitation tool. However, few studies evaluated the potential effects of exercise training to induce anticarcinogenic effects in the plasma of older adults (Soares et al., 2021). Furthermore, to date, no study focused on mitochondrial activity of cancer cells after exercised plasma treatment. This *in vitro* study evaluated the role of the older adult plasma submitted to multimodal exercise training in the viability and proliferation of immortalized prostate cancer androgen unresponsive PC3 cells.

## Methods

### Participants

Eight institutionalized older adults (aged 73.38 ± 11.28 y; body mass index 27.8 ± 4.9 kg/m^2^) living in a long-term facility in Porto Alegre City, south of Brazil, were considered. The inclusion and exclusion criteria were previously reported (Fraga et al., 2021). The sample size of older adults was limited to the individuals who lived in a single long-term facility and were able to perform exercise. Participants were not engaged in structured exercise training protocols for a period prior to 6 months before the trial. The Ethics Research Committee of Centro Universitario Metodista-IPA, Brazil, approved the current study number 3.376.078. All participants signed written informed consent before enrollment, and all procedures were in conformity with the Declaration of Helsinki. This study was not prospectively registered. Additionally, we collected venous blood samples from young lean men with high physical fitness (PF) (high PF: *n* = 5; age, 27.8 ± 5.6 y; body mass index, 24.3 ± 1.3 kg/m^2^; VO_2Peak_ 49.7 ± 2.67 ml.kg.min) and low PF (low PF: *n* = 5; age, 29.8 ± 2.7 y; body mass index, 23.1 ± 2.1 kg/m^2^; VO_2Peak_ 37.8 ± 3.1 ml.kg.min) as controls of the study. The criteria for being physically active were the completion of at least 3 h of endurance training per week, for a minimum of 3 years, and a peak oxygen consumption (VO_2Peak_) of at least 45 ml/kg/min. Individuals who reported less than 100 min of physical activity per week and not engaging in regular exercise training for at least 2 years were defined as physically inactive/sedentary. All young participants were recruited from the general community of Porto Alegre/Brazil and performed a cardiopulmonary exercise (CPET) to determine their physical fitness. Detailed information regarding recruitment criteria was previously published in Dorneles et al. (2019). Physical fitness was evaluated by a CPET test as previously described Dorneles et al., 2019. In brief, the CPET was performed on an electric treadmill (Centurion 300; Micromed, Brasilia, Brazil) using a ramp protocol. Both the speed and incline of the treadmill gradually increased up to the maximum limit of the participant. Ventilatory and metabolic parameters were collected by respiration using a Metalyzer 3B (Cortex, Leipzig, Germany) and were analyzed after the mean of the data in eight respiratory cycles. The CPET system was calibrated before each test with respect to both airflow and O_2_ and CO_2_ analyzers. The average of the last 30 s of the test was used to determine the VO_2_ peak.

### Study Design and Training Protocol

Older adults participated in a multimodal exercise training (8 weeks, 2x/week, 60 min each session) supervised by a trained physiotherapist. The multimodal protocol used in the current study was based on a previous study (Pereira et al., 2018). The components of intervention are in adherence to the Consensus on Exercises Reporting Template (CERT) (Supplementary Material S1). The exercise intervention was intended to stimulate physiological, perceptual, and cognitive mechanisms. Therefore, the exercise sessions were planned to guarantee stimuli relevant to promoting simultaneous motor and cognitive stimulation and to incorporate challenging activities that induced the participants to mobilize several types of abilities.

Specifically, each session was divided into the four following moments: 1) warm-up (5 min), with stretching and active upper/lower limb exercises; 2) exercises focused on cardiovascular capacity, strength, balance/agility, and flexibility (25 min), which included walking, stationary gait, resistance exercises for the main upper/lower muscle groups, unipodal support with open/closed arms over the chest, anterior/lateral inclination, and static stretches; 3) exercises focused on perception and cognition (such as double-task), attention, memory, and processing of requested actions (25 min), such as walking and naming fruit/color names, completing previously established circuits, attending to requested verbal commands, and memorizing motor/verbal signals; and 4) stretching, breathing, and relaxation movements to cool down (5 min).

The sessions aimed to develop a safe and progressive training schedule and the level of difficulty of the proposed tasks increased along the program. Exercise intensity and tolerability were supervised by observation, with an emphasis on participants’ respiratory responses to talk during exercise performance. In cases where the exercise intensity was not perceived as tolerable by the participants, the intensity was reduced until being perceived as comfortable (Marmeleira et al., 2018).

### Blood Samples and Cytokine Measurement

Fasting venous blood samples were collected from the antecubital vein into EDTA tubes (8 ml), centrifuged (1,000 *g*, 10 min), aliquoted into microtubes, and stored at -80°C. Blood collection was performed before the first exercise session and 48 h after the last exercise bout. We selected a panel of cytokines related to metabolic, endocrine, and immunological effects that are impacted by aging. The systemic levels of interleukin (IL)-1ra, IL-1β, IL-2, IL-6, IL-10, IL-17, interferon (IFN)-α, tumor necrosis factor (TNF)-α (all from Thermo Fisher, United States), fibroblast growth factor (FGF)-1, platelet-derived growth factor (PDGF), and transforming growth factor (TGF)-α (all from RayBiotech, United States) were determined by enzyme-linked immunosorbent assay (ELISA) following the manufacturer’s instructions in a microplate reader (EzReader, Biochrom, United States). The coefficients of variation of all assays were always <7.5%.

### Cell Culture Experiments

The PC3 prostate cancer cell line from the American Type Culture Collection (ATCC^®^ CRL-1435™) was used in this study. PC3 cells were used in this study due to their use in investigating biochemical changes in advanced prostate cancer cells and their characteristic of androgen unresponsiveness which makes them ideal for aging studies. Cells were cultured in 75 cm^2^ flasks using Roswell Park Memorial Institute medium-1640 (RPMI-1640) supplemented with 10% (v/v) fetal bovine serum (FBS), 0.1 mg/ml streptomycin, and 100 U/mL penicillin. Cells were maintained in a humidified incubator at 37°C and at 5% CO_2_, during a maximum of 15 passages. Cells were plated in a 96-well plate with 10% FBS for 24 h before replacing the FBS with human plasma. During experiments, 10% FBS was replaced with 10% of human plasma obtained from older adults before or after the training sessions and from young lean men with low and high PF. Plasma obtained before and after training was incubated separately with PC3 cells. All experiments were done in triplicate, and the results were shown as the mean of triplicates from three independent experiments.

After a 48-h treatment, the cell viability assay [3-(4,5-dimethylthiazole bromide-2-yl)-2,5-diphenyltetrazolium bromide (MTT) and LDH release] was performed by colorimetric reduction of MTT to formazan. Samples were read using a spectrophotometer at 492 nm (Mosmann, 1983). Appropriate controls with dimethyl sulfoxide (DMSO) (extract solvent) and the blank liposome were performed to eliminate the membrane solvent hydrolysis effect in the interpretation of the results. PC3 cells treated with pre-post-training plasma obtained from older adults were also incubated with N-acetylcysteine (NAC, 2 mM) for 24 h before the cell viability determination by MTT.

LDH activity was evaluated in a commercial kit (LDH Roche, Brazil). Released LDH in the culture media was coupled to an enzymatic assay yielding a red color, the intensity of which was measured at 490 nm using a microplate reader.

The proliferative response of PC3 was evaluated by the decay of carbofluorescein succinimidyl ester (CFSE) fluorescence using an FACSCalibur (Becton Dickinson, San Jose, CA) flow cytometer equipped with a blue argon laser (488 nm) and a 530/30 nm bandpass filter. The CFSE fluorescence was analyzed in histograms of the FL1 channel. The “M1 region” was defined as CFSE-stained cells derived from unstimulated cultures, which represented the peak of quiescent cells, and the M2 region was defined as proliferative cells according to the peaks of CFSE intensity. Apoptosis and necrosis of PC3 cells were measured using FITC Annexin V with a propidium iodide Apoptosis/necrosis Detection Kit (556547, BD Biosciences) according to the manufacturer’s instructions using an FACSCalibur flow cytometer (BD Biosciences).

Mitochondrial membrane polarization and cytosolic and mitochondrial reactive oxygen species (ROS) analyses were performed after 12 h of *in vitro* PC3 incubation with pre- and post-exercise training plasma. The mitochondrial membrane potential (ΔΨm) was quantified according to a method previously described (Ferlini and Scambia, 2007) using the fluorescent dye rhodamine 123 (Rh 123, Sigma-Aldrich, United States). Mitochondrial superoxide generation in live cells was assessed with MitoSOX Red (Invitrogen, Thermo Fisher, United States). Cytosolic ROS production was evaluated using the reagent 20,70-dichlorofluorescein diacetate (DCF-DA), which becomes fluorescent when oxidated by ROS (Sigma Aldrich, United States). Analyses were performed by using CELLQuest Pro Software (BD Bioscience) on an FACSCalibur flow cytometer (BD Bioscience).

### Statistical Analysis

Data were analyzed in GraphPad Prism 8.0 (United States). Data were presented as mean ± standard deviation. Nonparametric tests were used in this study because of the small sample size of *in vitro* experiments. The before–after exercise training comparisons were analyzed by the Wilcoxon matched-pairs signed rank test. The effects of plasma obtained from high PF and low PF as well as the plasma collected before and after exercise training from older adults on cell viability evaluated by the MTT assay, LDH release, and PC3 cell proliferation were compared by the Kruskal–Wallis test corrected by Dunn’s post-test for multiple comparisons. Spearman’s correlation test was applied to verify the relationship between systemic inflammatory mediators and *in vitro* PC3 measurements. *p*-value ≤0.05 was considered statistically significant.

## Results

### Prostate Cancer Cell Viability and Proliferation Are Modulated by Physical Fitness Status and Exercise Training Practice

**FIGURE 1:**
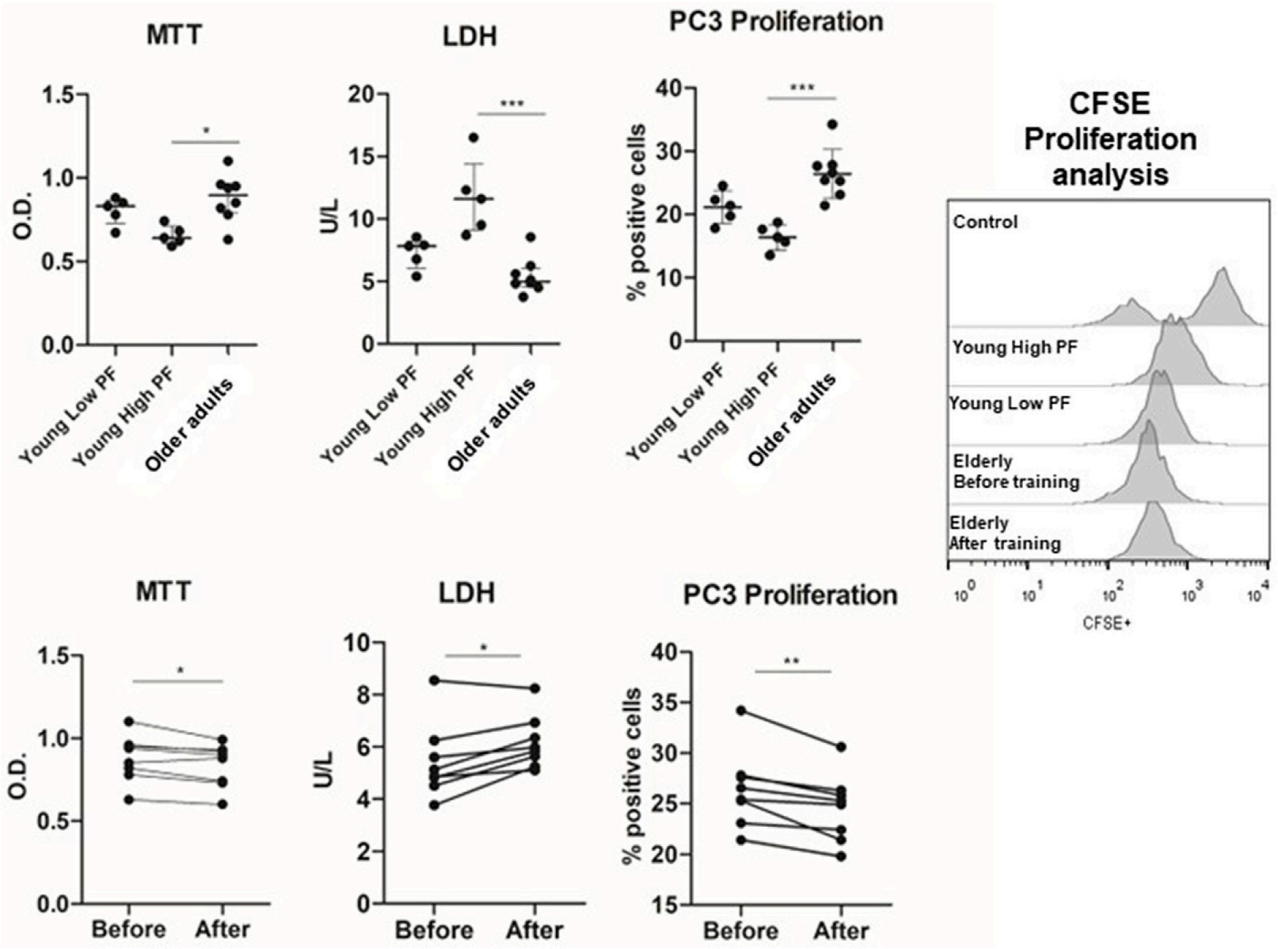
Effects of plasma from young high-PF adults (*n* = 5), young low-PF adults (*n* = 5), and older adults (*n* = 8) submitted to multimodal exercise training on viability and proliferation of the PC3 prostate cancer cell line. PC3 prostate cancer cell lines were incubated with the plasma of young high-PF adults and young low-PF adults and with the plasma of institutionalized older adults obtained before training and 48 h after 8 weeks of exercise training. Cell viability was evaluated by MTT and LDH activity and proliferation by the drop of CFSE fluorescence in PC-3 cells after 48 h of cell culture. All experiments were done in triplicate, and the results are shown as the mean of triplicates from three independent experiments. *p < 0.05; **p < 0.01; ***p < 0.001.

**FIGURE 2:**
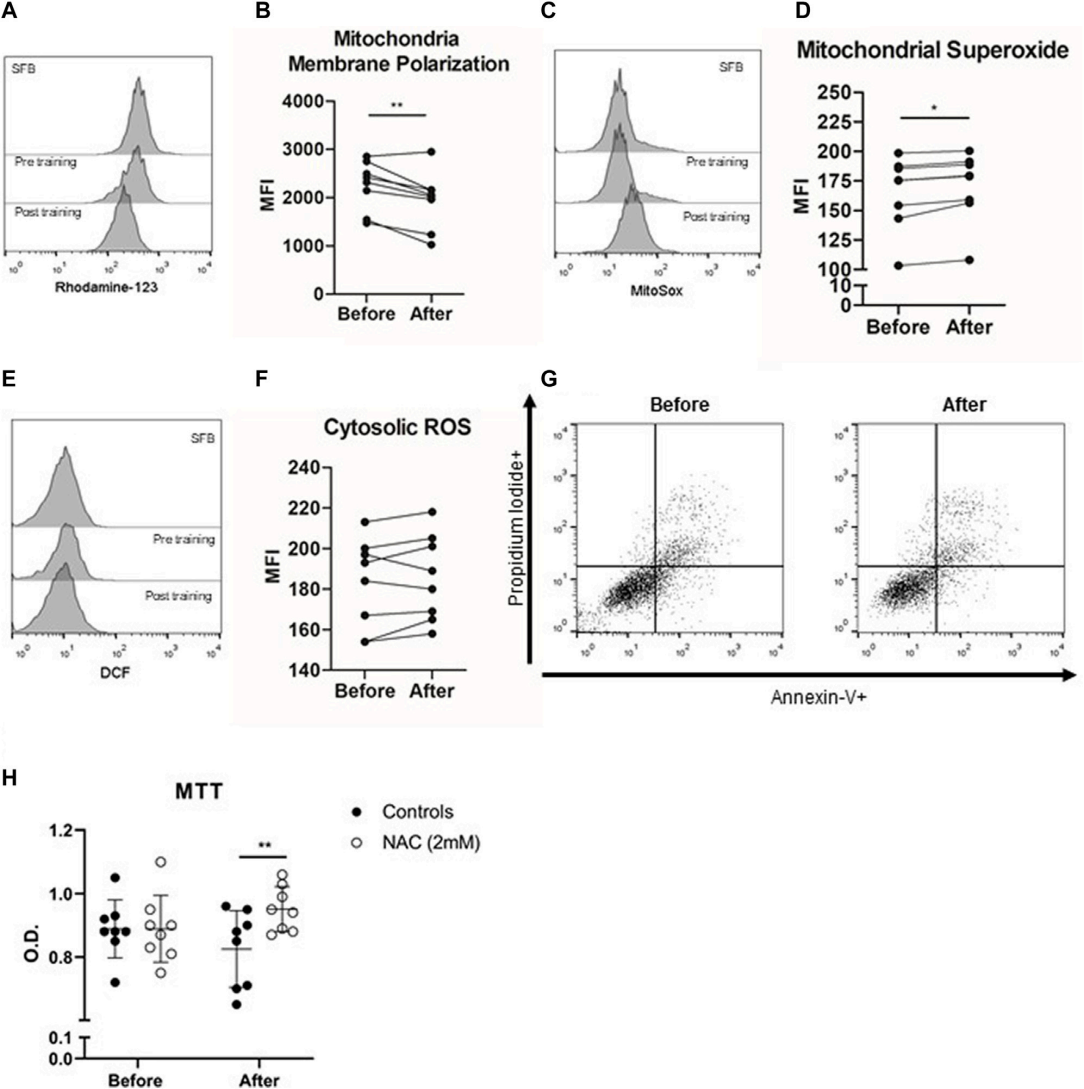
Anticarcinogenic effects of plasma from trained older adults (*n* = 8) in the PC3 prostate cancer cell line. PC-3 Prostate cancer cell lines were incubated with the plasma of institutionalized older adults obtained before training and 48 h after 8 weeks of exercise training. The histogram of rhodamine-123 **(A)** evaluated the mitochondrial membrane potential **(B)**, the histogram of Mitosox **(C)** quantified mitochondrial superoxide **(D)** generation, and the histogram of DCF **(E)** evaluated cytosolic ROS **(F)** production after 12 h of PC3 cell line incubation with plasma obtained before and after exercise training. Apoptosis (Annexin-V+ cells) or necrosis (propidium iodide + cells) did not differ before and after comparison **(G)**. **(H)** Cell viability (MTT) was evaluated without and with NAC concomitant with the incubation of PC3 cells with plasma from older adults. All experiments were done in triplicate, and the results are shown as the mean of triplicates from three independent experiments. *p < 0.05; **p < 0.01; ***p < 0.001.

All participants completed all intervention trial sessions, and no adverse effect was reported. Furthermore, all participants provide blood samples before and after exercise training. First, we evaluated the effects of plasma obtained from young individuals with different PF statuses (low and high) and from older adults before the engagement in a multimodal exercise training (Figures 1, 2). PC3 incubated with plasma acquired from young men with high PF presented low cell viability (*p* = 0.01) and higher LDH released (*p* = 0.001) compared to the incubation trial with plasma obtained from the older adults. The percentage of cells undergoing proliferation was lower in the incubation of PC3 with plasma from the high-PF group compared to the older adults (*p* = 0.001). However, the analyses of PC3 cell viability or proliferation in the low-PF young group did not differ compared to the other groups (*p* > 0.05).

Thus, plasma factors in the blood of individuals with a higher PF status presented anticarcinogenic effects in a prostate cancer cell line compared to those observed in the plasma of older adults. Next, older adults were submitted to 8 weeks of multimodal exercise training, and plasma was obtained after the intervention. Interestingly, the incubation of the PC3 prostate cancer cell line with the post-training plasma of older adults resulted in lower cell viability (*p* = 0.03) and cell proliferation (*p* = 0.007) and higher LDH release (*p* = 0.02) compared to the pre-training plasma condition.

### Plasma Acquired After Multimodal Exercise Training Changes the Mitochondria Membrane Potential and Increases Mitochondrial Reactive Oxygen Species Production

Next, we evaluated the role of mitochondria-induced ROS generation in the anticarcinogenic effects of plasma of trained older adults. After 12 h of PC3 cell culture, post-training plasma decreased mitochondrial membrane polarization compared to pre-training plasma incubation conditions (*p* < 0.01). The mitochondrial membrane depolarization was accompanied by increased mitochondrial ROS production in the PC3 cell line incubated with post-training plasma (*p* = 0.007), without changes in cytosolic ROS generation (*p* > 0.05). Finally, PC3 cell line incubation with post-training plasma did not change apoptosis or necrosis cell events (*p* > 0.05). In addition, we treated PC-3 prostate cancer cells with NAC, an antioxidant molecule and a glutathione/trypanothione precursor, to evaluate if the viability of PC3 cells could be preserved. When we added NAC in the exercised plasma treatment of PC3 cells, the action of conditioned plasma was almost completely reversed (*p* = 0.038).

### Multimodal Exercise Training Alters Systemic Cytokine Levels of Institutionalized Older Adults

**FIGURE 3:**
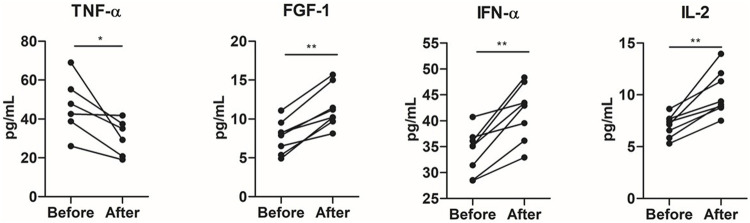
Impact of 8 weeks of multimodal exercise training on systemic cytokine levels of institutionalized older adults. *p < 0.05; **p < 0.01; ***p < 0.001.

Increases in the plasma levels of interleukin (IL)-2 (*p* = 0.007), interferon-alpha (IFN-α) (*p* = 0.007), and fibroblast growth factor (FGF)-1 (*p* = 0.001) occurred after the training period. On the other hand, tumor necrosis factor-alpha (TNF-α) concentrations decreased (*p* = 0.03) after training (Figure 3). No statistical differences were observed in IL-1ra, IL-1β, IL-6, IL-10, PDGF, IL-17a, or TGF-α (*p* > 0.05) (Supplementary Figure S2).

The percentage (%) of change of cytokine plasma levels in response to multimodal exercise training was correlated with the percentage of changes of *in vitro* experiments, MTT, LDH, CFSE cell proliferation, cytosolic ROS, mitochondrial membrane polarization, and mitochondrial ROS, in the PC3 prostate cancer cell line. IL-10 correlated with mitochondrial membrane polarization (r = 0.82; *p* = 0.04), TNF-α inversely correlated with mitochondrial ROS (r = −0.94; *p* = 0.005), IL-6 positively correlated with mitochondrial ROS (r = 0.82; *p* = 0.04), and cell viability evaluated by MTT correlated with IL-17 (r = 0.76; *p* = 0.02).

## Discussion

The main results of this study were as follows: 1) the incubation of the PC3 prostate cancer cell line with plasma acquired from young men with high PF leads to lower cell viability and proliferation compared to the cell treatment with pre-training plasma obtained from older adults; 2) post-exercise training plasma of older adults leads to lower cell viability and proliferation rates in PC3 prostate cancer cells; 3) conditioned post-training plasma induced mitochondrial membrane depolarization and higher mitochondrial ROS, but not cytosolic ROS, in PC3 prostate cancer cells without changes in apoptosis/necrosis rate; 4) 8 weeks of multimodal exercise training increases the systemic levels of IL-2, IFN-α, and FGF-1 and decreases the TNF-α concentrations in aged individuals. Taken together, we showed for the first time that multimodal exercise training induces systemic inflammatory adaptations in institutionalized older adults in parallel to the enhanced anticarcinogenic potential of blood mediators against prostate cancer through changes in mitochondrial membrane polarization and mitochondrial ROS generation.

The plasma collected from highly conditioned young men, but not from low-PF individuals, decreased the PC3 cell viability and lowered cell proliferation. These results are in line with the results previously reported by a series of systematic reviews and meta-analysis recently published, which demonstrated the anticarcinogenic potential of peripheral blood factors of exercised individuals (Metcalfe et al., 2021; Soares et al., 2021). The regular practice of exercise leads to chronic adaptations that reduce cancer risk through changes in cancer risk circulating biomarkers (Friedenreich et al., 2017). The difference between young and older adults might also occur due to the age-related changes in immune functions that contribute to the relationship concerning cancer and the aging process (Ames and Gold, 1991). The mechanisms of cancer development with age involve the chronic elevations in concentrations of pro-inflammatory cytokines resulting in low-grade inflammation together with a lifetime accumulation of DNA mutation, leading to inevitable errors during repair or replication of damaged DNA (López-Otín et al., 2013; Ferrucci and Fabbri, 2018). Notwithstanding, the present data reinforce the concept that plasma from young trained human participants can modulate cancer cell viability and proliferation and can be applied to probe mechanisms and countermeasures to age-related cancer development.

Interestingly, apoptosis and necrosis rates were unchanged after the incubation of the PC3 prostate cell line with conditioned exercised plasma, confirming previous data that demonstrate that exercised mediators reduce cell viability without changes in cell death pathways (Rundqvist et al., 2013; Matos et al., 2021). Notably, several studies were conducted using acute exercise session models, and the tumor-suppressive effects of chronic exercise training are poorly studied. Moreover, some past longitudinal studies contrast with our findings regarding the role of plasma collected after exercise training on changes in cancer cell viability and proliferation (Devin et al., 2019; Soares et al., 2021). In a recent systematic review with meta-analysis, Soares and others (2021) identified that the viability of cancer cells did not change after post-training plasma stimuli. The authors indicated that conflicting results may be related to the population study, cancer cell lineage, or exercise intervention types. On the other hand, prostate cancer cells may be more susceptible to changes in post-training plasma composition. In this line, plasma acquired from trained rats reduced prostate cancer cell viability without changes in other key prostate tumor characteristics (i.e., migration and cell cycle) (Opoku-Acheampong et al., 2019). Similarly, two recent studies from the same research group demonstrated that long-term supervised multimodal exercise training changes blood factor composition and the incubation of cancer cells with the trained serum reduces tumor cell growth (Kim J.-S. et al., 2022; Kim JS. et al., 2022).

Here, we describe for the first time the mitochondrial dysfunction in the PC3 prostate cell line incubated with post-exercise training plasma of older adults. Targeting cancer cell mitochondria has been long suggested as a therapeutic approach to control cell proliferation and growth (Chattopadhyay and Roy, 2017). In this sense, several pharmacological therapies alter mitochondrial functions to induce cell death and lower tumor progression (Wen et al., 2013). In the present study, we show that depolarization of the mitochondrial membrane potential is associated with increasing superoxide production (mitochondrial ROS) after 12 h of PC3 incubation with the conditioned plasma of the older adults. Furthermore, mitochondrial membrane depolarization leads to translocation of apoptosis-induced factor (AIF) to the nuclei and activation of caspase-12 associated with the endoplasmic reticulum to induce cell death (Wang and Youle, 2009). Moreover, mitochondrial membrane depolarization directly affects complex II and its function in electric chain transport, leading to ROS generation and the activation of the apoptotic cascade (Zorov et al., 2014). Furthermore, increases in p53 protein expression in prostate tumor cells by exercise conditioned serum were previously related to a reduction in cell growth and proliferation (Leung et al., 2004). P53 is commonly described as a tumor suppressor gene by its role in conserving genome stability and preventing DNA mutation and becomes activated in response to myriad stressors, including oxidative stress (Leung et al., 2004). However, the lack of changes in apoptosis rate after conditioned plasma incubation may indicate the need for repeated or prolonged incubation time to induce cancer cell death. Notably, decreases in mitochondria membrane depolarization result in elevated cytochrome c release, a marker of low cell viability, since proper levels of cellular ATP and redox balance are essential for cell viability and proliferation (MacDonald et al., 2018). Furthermore, pharmacological suppression of NADPH oxidase (NOX), an enzymatic complex capable of oxidizing NAPH or NADH to NADP+ or NAD+, directly impacts cancer cell mitochondria, leading to a decrease in cellular glycolysis and a loss of cell viability and growth (Lu et al., 2012). Furthermore, we cannot exclude the fact that mitochondrial membrane depolarization and ROS generation may directly impact on cell viability and proliferation by targeting the Warburg effect, an event related to increases in aerobic glycolysis that supports tumor growth (Wen et al., 2013).

Here, exercise training was able to decrease pro-inflammatory TNF-α levels, suggesting a role to induce an anti-inflammatory profile in institutionalized elderlies. However, other classic proinflammatory mediators, such as IL-6, IL-17a, and IL-1β, did not change after 8 weeks of multimodal exercise. These results may suggest that the potential anti-inflammatory adaptations observed in previous observational and exercise training studies (Sellami et al., 2021) may need a longer intervention time than 8 weeks to be achieved. On the other hand, this is the first study to observe increased FGF-1 increased after an exercise training period. FGF-1, also called acidic FGF, plays an important role in the regulation of cell survival, cell division, angiogenesis, cell differentiation, and migration (Li, 2018). Interestingly, experimental studies show that mice treated with FGF-1 restore blood glucose levels and endothelial functions, highlighting the role of this growth factor in vascular health and metabolic control (Keeley et al., 2019). Furthermore, the mutated *fgf1* gene is linked to an accelerated neurological senescence profile in mice (Carter et al., 2005). Thus, FGF-1 emerges as an important biological mediator in the control of aging through exercise training.

We found an increase in IL-2 and IFN-α levels in the peripheral blood of the older adults after the exercise training period. Both IL-2 and IFN-α have strong antitumorigenic direct effects against cancer cells, and *in vitro* cytokine treatment of prostate tumor cell lines can effectively alter a number of prostate carcinoma properties closely associated with tumor invasion and the metastatic phenotype (Westdorp et al., 2014). IFN has an important role in the regulation of mitochondrial functions, and seminal studies pointed out that IFN treatment causes a reduction in cellular ATP levels and inhibits tumor growth (Shan, Vazquez, Lewis, 1990; Lewis et al., 1996). Furthermore, IFN type I has a cross-over interaction with mitochondrial ROS to control cell proliferation and survival (Yim et al., 2012; Wang et al., 2017). The correlation between post-training cytokine levels and PC3 cell viability, proliferation, and mitochondrial functions revealed some associations between changes in systemic inflammatory mediators and the cancer cell phenotype. In this line, a recent study conducted by Orange et al. (2022) indicated that systemic myokine release induced by exercise directly impacts tumor cell DNA damage induction and repair which may be associated with reductions in cancer cell proliferation.

In conclusion, this exercise training study described for the first time the potential of conditioned plasma to decrease cell viability and proliferation in the PC3 prostate tumor cell line. We also demonstrated a new mechanistic pathway by which exercise may alter prostate cell functions through mitochondrial functions, mainly by mitochondrial membrane depolarization and superoxide formation. These changes were accompanied by alterations in several systemic inflammatory mediators after multimodal exercise training. Collectively, changes in blood factor composition by exercise training contribute to the control of prostate tumorigenesis, suggesting the role of exercise as an adjuvant therapy in cancer treatment and prevention.

## Data Availability

The raw data supporting the conclusion of this article will be made available by the authors, without undue reservation.

## References

[B1] Ames B. N. Gold L. S. (1991). Endogenous Mutagens and the Causes of Aging and Cancer. Mutat. Res. 250 (1–2), 3–16. 10.1016/0027-5107(91)90157-J 1944345

[B2] Barbé-Tuana F. Funchal G. Schmitz C. R. R. Maurmann R. M. Bauer M. E. (2020). The Interplay between Immunosenescence and Age-Related Diseases. Semin. Immunopathol. 42 (5), 545–557. 10.1007/S00281-020-00806-Z 32747977 PMC7398288

[B3] Bauer M. E. De la Fuente M. (2014). Oxidative Stress, Inflammaging, and Immunosenescence. Inflamm. Adv. Age Nutr. Res. Clin. Interventions 2014, 39–47. 10.1016/B978-0-12-397803-5.00004-6

[B4] Boland M. L. Chourasia A. H. Macleod K. F. (2013). Mitochondrial Dysfunction in Cancer. Front. Oncol. 3, 292. 10.3389/FONC.2013.00292 24350057 PMC3844930

[B5] Carter T. A. Greenhall J. A. Yoshida S. Fuchs S. Helton R. Swaroop A. (2005). Mechanisms of Aging in Senescence-Accelerated Mice. Genome Biol. 6 (6), R48. 10.1186/GB-2005-6-6-R48 15960800 PMC1175968

[B6] Chattopadhyay E. Roy B. (2017). Altered Mitochondrial Signalling and Metabolism in Cancer. Front. Oncol. 7, 43. 10.3389/fonc.2017.00043 28373964 PMC5357656

[B7] Devin J. L. Hill M. M. Mourtzakis M. Quadrilatero J. Jenkins D. G. Skinner T. L. (2019). Acute High Intensity Interval Exercise Reduces Colon Cancer Cell Growth. J. Physiol. 597, 2177–2184. 10.1113/JP277648 30812059 PMC6462486

[B8] Dorneles G. P. Silva I. Boeira M. C. Valentini D. Fonseca S. G. Dal Lago P. (2019). Cardiorespiratory Fitness Modulates the Proportions of Monocytes and T Helper Subsets in Lean and Obese Men. Scand. J. Med. Sci. Sports 29, 1755–1765. 10.1111/sms.13506 31241790

[B9] Ferlini C. Scambia G. (2007). Assay for Apoptosis Using the Mitochondrial Probes, Rhodamine123 and 10-N-Nonyl Acridine Orange. Nat. Protoc. 2, 3111–3114. 10.1038/nprot.2007.397 18079710

[B10] Ferrucci L. Fabbri E. (2018). Inflammageing: Chronic Inflammation in Ageing, Cardiovascular Disease, and Frailty. Nat. Rev. CardiolCardiology 15 (9), 505–522. 10.1038/S41569-018-0064-2 PMC6146930 30065258

[B11] Fraga I. Elsner V. Weber C. Galiano W. Iraci L. Wohlgemuth M. (2021). Effects of a Multimodal Exercise Protocol on Functional Outcomes, Epigenetic Modulation and Brain-Derived Neurotrophic Factor Levels in Institutionalized Older Adults: A Quasi-Experimental Pilot Study. Neural Regen. Res. 16 (12), 2479–2485. 10.4103/1673-5374.313067 33907037 PMC8374571

[B12] Friedenreich C. M. Shaw E. Neilson H. K. Brenner. D. R. (2017). Epidemiology and Biology of Physical Activity and Cancer Recurrence. J. Mol. Med. 95 (10), 1029–1041. 10.1007/S00109-017-1558-9 28620703 PMC5613065

[B13] Hwang J. H. McGovern J. Minett G. M. Della Gatta P. A. Roberts L. Harris J. M. (2020). Mobilizing Serum Factors and Immune Cells through Exercise to Counteract Age-Related Changes in Cancer Risk. Exerc Immunol. Rev. 26, 80–99. 32139350

[B14] Jana S. Patra K. Sarkar S. Jana J. Mukherjee G. Bhattacharjee S. (2014). Antitumorigenic Potential of Linalool Is Accompanied by Modulation of Oxidative Stress: An *In Vivo* Study in Sarcoma-180 Solid Tumor Model. Nutr. Cancer 66 (5), 835–848. 10.1080/01635581.2014.904906 24779766

[B15] Keeley T. Kirov A. Koh W. Y. Demambro V. Bergquist I. Cotter J. (2019). Resistance to Visceral Obesity Is Associated with Increased Locomotion in Mice Expressing an Endothelial Cell‐specific Fibroblast Growth Factor 1 Transgene. Physiol. Rep. 7 (7), e14034. 10.14814/PHY2.14034 30972920 PMC6458108

[B16] Kim J. S. Taaffe D. R. Galvão D. A. Hart N. H. Gray E. Ryan C. J. (2022b). Exercise in Advanced Prostate Cancer Elevates Myokine Levels and Suppresses Iin-Vvitro Cell Growth. Prostate Cancer Prostatic Dis. 25 (1), 86–92. 10.1038/s41391-022-00504-x 35152272 PMC8853098

[B17] Kim J.-S. Galvão D. A. Newton R. U. Gray E. Taaffe D. R. (2021). Exercise-Induced Myokines and Their Effect on Prostate Cancer. Nat. Rev. Urol. 18, 519–542. 10.1038/S41585-021-00476-Y 34158658

[B18] Kim J.-S. Wilson R. L. Taaffe D. R. Galvão D. A. Gray E. Newton R. U. (2022a). Myokine Expression and Tumor-Suppressive Effect of Serum after 12 Wk of Exercise in Prostate Cancer Patients on ADT. Med. Sci. Sports Exerc 54, 197–205. 10.1249/MSS.0000000000002783 34559721 PMC8754092

[B19] Leung P.-S. Aronson W. J. Ngo T. H. Golding L. A. Barnard R. J. (2004). Exercise Alters the IGF axis *In Vivo* and Increases P53 Protein in Prostate Tumor Cells *In Vitro* . J. Appl. Physiology 96, 450–454. 10.1152/japplphysiol.00871.2003 14715676

[B20] Lewis J. A. Huq A. Najarro P. (1996). Inhibition of Mitochondrial Function by Interferon. J. Biol. Chem. 271, 13184–13190. 10.1074/jbc.271.22.13184 8662694

[B21] Li X. (2018). FGFs in Injury Repair and Regeneration. Fibroblast Growth Factors 2018, 17–144. 10.1016/B978-0-12-816142-5.00002-3

[B22] López-Otín C. Blasco M. A. Partridge L. Serrano M. Kroemer G. (2013). The Hallmarks of Aging. Cell 153 (6), 1194–1217. 10.1016/J.CELL.2013.05.039 23746838 PMC3836174

[B23] Lu W. Hu Y. Chen G. Chen Z. Zhang H. Wang F. (2012). Novel Role of NOX in Supporting Aerobic Glycolysis in Cancer Cells with Mitochondrial Dysfunction and as a Potential Target for Cancer Therapy. PLoS Biol. 10 (5), e1001326. 10.1371/journal.pbio.1001326 22589701 PMC3348157

[B24] MacDonald J. A. Kura N. Sussman C. Woods D. C. (2018). Mitochondrial Membrane Depolarization Enhances TRAIL-Induced Cell Death in Adult Human Granulosa Tumor Cells, KGN, through Inhibition of BIRC5. J. Ovarian Res. 11, 89. 10.1186/s13048-018-0463-3 30326924 PMC6192357

[B25] Marmeleira J. Galhardas L. Raimundo A. (2018). Exercise Merging Physical and Cognitive Stimulation Improves Physical Fitness and Cognitive Functioning in Older Nursing Home Residents: a Pilot Study. Geriatr. Nurs. 39, 303–309. 10.1016/j.gerinurse.2017.10.015 29221898

[B26] Matos B. Patrício D. Henriques M. C. Freitas M. J. Vitorino R. Duarte I. F. (2021). Chronic Exercise Training Attenuates Prostate Cancer-Induced Molecular Remodelling in the Testis. Cell Oncol. 44 (2), 311–327. 10.1007/S13402-020-00567-9 PMC12980742 33074478

[B27] Metcalfe R. S. Kemp R. Heffernan S. M. Churm R. Chen Y. C. Ruffino J. S. (2021). Anti-Carcinogenic Effects of Exercise-Conditioned Human Serum: Evidence, Relevance and Opportunities. Eur. J. Appl. Physiology 121, 8. 10.1007/S00421-021-04680-X PMC8260517 33864493

[B28] Mosmann T. (1983). Rapid Colorimetric Assay for Cellular Growth and Survival: Application to Proliferation and Cytotoxicity Assays. J. Immunol. Methods 65, 55–63. 10.1016/0022-1759(83)90303-4 6606682

[B29] Opoku-Acheampong A. B. Baumfalk D. R. Horn A. G. Kunkel O. N. Ganta C. K. McCullough D. J. (2019). Prostate Cancer Cell Growth Characteristics in Serum and Prostate-Conditioned Media from Moderate-Intensity Exercise-Trained Healthy and Tumor-Bearing Rats. Am. J. Cancer Res. 9, 650–667. 31105994 PMC6511645

[B30] Orange S. T. Jordan A. R. Odell A. Kavanagh O. Hicks K. M. Eaglen T. (2022). Acute Aerobic Exercise‐conditioned Serum Reduces Colon Cancer Cell Proliferation *In Vitro* through Interleukin‐6‐induced Regulation of DNA Damage. Intl J. Cancer 151 (2), 265–274. 10.1002/ijc.33982 PMC9314683 35213038

[B31] Orange S. T. Jordan A. R. Saxton J. M. (2020). The Serological Responses to Acute Exercise in Humans Reduce Cancer Cell Growth *In Vitro*: A Systematic Review and Meta‐analysis. Physiol. Rep. 8, 22. 10.14814/PHY2.14635 PMC7673630 33207085

[B32] Pereira C. Rosado H. Cruz-Ferreira A. Marmeleira J. (2018). Effects of a 10-Week Multimodal Exercise Program on Physical and Cognitive Function of Nursing Home Residents: A Psychomotor Intervention Pilot Study. Aging Clin. Exp. Res. 30 (5), 471–479. 10.1007/S40520-017-0803-Y 28776280

[B33] Rundqvist H. Augsten M. Strömberg A. Rullman E. Mijwel S. Kharaziha P. (2013). Effect of Acute Exercise on Prostate Cancer Cell Growth. PloS One 8 (7), e67579. 10.1371/JOURNAL.PONE.0067579 23861774 PMC3702495

[B34] Sellami M. Bragazzi N. L. Aboghaba B. Elrayess M. A. (2021). The Impact of Acute and Chronic Exercise on Immunoglobulins and Cytokines in Elderly: Insights from a Critical Review of the Literature. Front. Immunol. 12, 1873. 10.3389/FIMMU.2021.631873 PMC8079972 33936044

[B35] Seo J. H. Agarwal E. Chae Y. C. Lee Y. G. Storaci A. M. Garlick D. S. (2019). Mitochondrial Fission Factor Is a Novel Myc-dependent Regulator of Mitochondrial Permeability in Cancer. EBioMedicine 48, 353–363. 10.1016/j.ebiom.2019.09.017 31542392 PMC6838406

[B36] Shan B. Vazquez E. Lewis J. A. (1990). Interferon Selectively Inhibits the Expression of Mitochondrial Genes: a Novel Pathway for Interferon-Mediated Responses. EMBO J. 9, 4307–4314. 10.1002/j.1460-2075.1990.tb07879.x 2176148 PMC552214

[B37] Soares C. M. Teixeira A. M. Sarmento H. Silva F. M. Rusenhack M. C. Furmann M. (2021). Effect of Exercise-Conditioned Human Serum on the Viability of Cancer Cell Cultures: A Systematic Review and Meta-Analysis. Exerc Immunol. Rev. 27, 24–41. 33965899

[B38] Taverna G. Seveso M. Giusti G. Hurle R. Graziotti P. Štifter S. (20142014). Senescent Remodeling of the Innate and Adaptive Immune System in the Elderly Men with Prostate Cancer. Curr. Gerontology Geriatrics Res. 2014, 1–11. 10.1155/2014/478126 PMC3977481 24772169

[B39] Wang C. Youle R. J. (2009). The Role of Mitochondria in Apoptosis. Annu. Rev. Genet. 43, 95–118. 10.1146/ANNUREV-GENET-102108-134850 19659442 PMC4762029

[B40] Wang Y. Yu X. Song H. Feng D. Jiang Y. Wu S. (2017). The STAT-ROS Cycle Extends IFN-induced C-ancer C-ell A-poptosis. Int. J. Oncol. 52, 305–313. 10.3892/ijo.2017.4196 29115415

[B41] Wen S. Zhu D. Huang P. (2013). Targeting Cancer Cell Mitochondria as a Therapeutic Approach. Future Med. Chem. 5 (1), 53–67. 10.4155/FMC.12.190 23256813 PMC3587793

[B42] Westdorp H. Sköld A. E. Snijer B. A. Franik S. Mulder S. F. Major P. P. (2014). Immunotherapy for Prostate Cancer: Lessons from Responses to Tumor-Associated Antigens. Front. Immunol. 5, 191. 10.3389/FIMMU.2014.00191 24834066 PMC4018526

[B43] Wu C.-L. Huang A.-C. Yang J.-S. Liao C.-L. Lu H.-F. Chou S.-T. (2011). Benzyl Isothiocyanate (BITC) and Phenethyl Isothiocyanate (PEITC)-mediated Generation of Reactive Oxygen Species Causes Cell Cycle Arrest and Induces Apoptosis via Activation of Caspase-3, Mitochondria Dysfunction and Nitric Oxide (NO) in Human Osteogenic. J. Orthop. Res. Official Publ. Orthop. Res. Soc. 29 (8), 1199–1209. 10.1002/JOR.21350 21374707

[B44] Xie K. Huang S. Dong Z. Gutman M. Fidler I. J. (1995). Direct Correlation between Expression of Endogenous Inducible Nitric Oxide Synthase and Regression of M5076 Reticulum Cell Sarcoma Hepatic Metastases in Mice Treated with Liposomes Containing Lipopeptide CGP 31362. Cancer Res. 55 (14), 3123–3131. (Accessed March 18, 2022). 7541713

[B45] Yim H. Y. Yang Y. Lim J.-S. Lee M. S. Zhang D.-E. Kim K. I. (2012). The Mitochondrial Pathway and Reactive Oxygen Species Are Critical Contributors to Interferon-Α/β-Mediated Apoptosis in Ubp43-Deficient Hematopoietic Cells. Biochem. Biophysical Res. Commun. 423, 436–440. 10.1016/j.bbrc.2012.05.154 PMC3389127 22683641

[B46] Zichri S. B. Kolusheva S. Shames A. I. Schneiderman E. A. Poggio J. L. Stein D. E. (2021). Mitochondria Membrane Transformations in Colon and Prostate Cancer and Their Biological Implications. Biochimica Biophysica Acta (BBA) - Biomembr. 1863 (1), 183471. 10.1016/J.BBAMEM.2020.183471 32931774

[B47] Zorov D. B. Juhaszova M. Sollott S. J. (2014). Magdalena Juhaszova, and Steven J. SollottMitochondrial Reactive Oxygen Species (ROS) and ROS-Induced ROS Release. Physiol. Rev. 94 (3), 909–950. 10.1152/PHYSREV.00026.2013 24987008 PMC4101632

