# Supplementary figures and images for: Potential Anticarcinogenic Effects From Plasma of Older Adults After Exercise Training: An Exploratory Study
Source: Front Physiol. 2022 Jul 6;13:855133. doi: 10.3389/fphys.2022.855133 (PMC9298496; doi:10.3389/fphys.2022.855133)

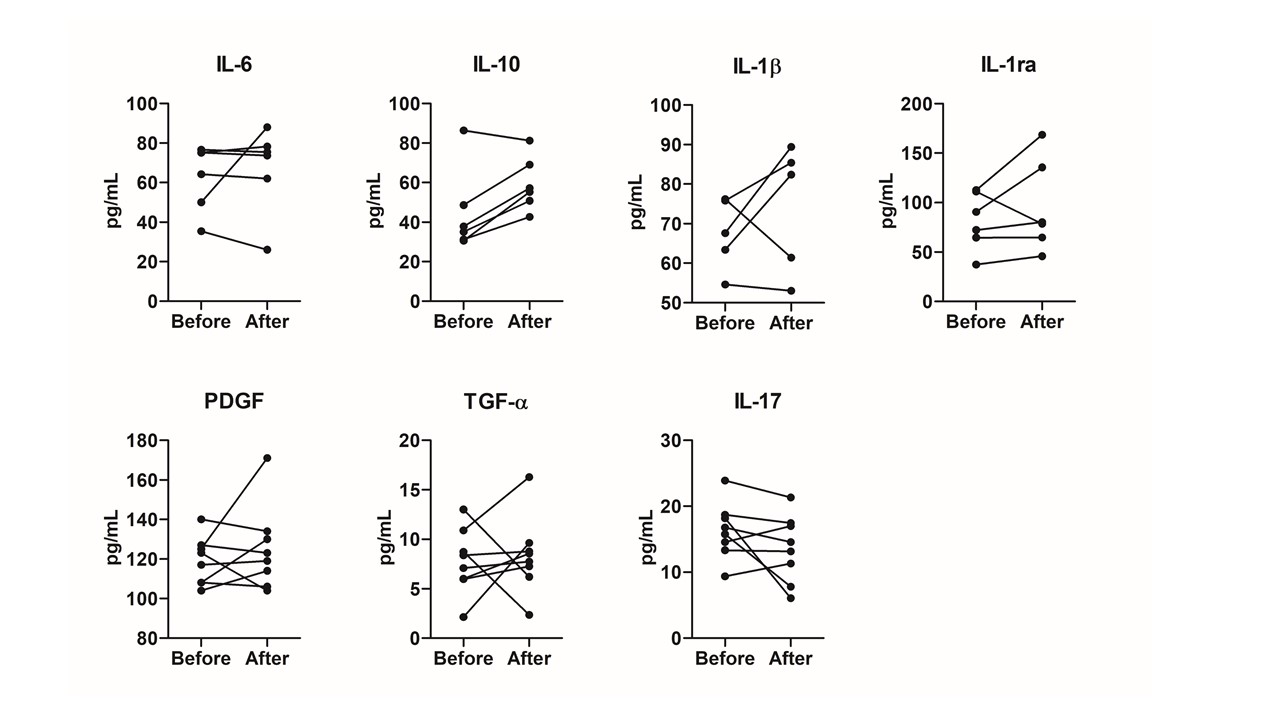

Supplement: Supplementary file 1 [file Image1.JPEG]
