# Supplementary material for: Potential Anticarcinogenic Effects From Plasma of Older Adults After Exercise Training: An Exploratory Study
Source: Front Physiol. 2022 Jul 6;13:855133. doi: 10.3389/fphys.2022.855133 (PMC9298496; doi:10.3389/fphys.2022.855133)
Supplement: Supplementary file 2 [file DataSheet1.PDF]

CERT ✓ Consensus on Exercise Reporting Template

A Checklist for what to include when reporting exercise programs

Section/Topic	Item #	Checklist item	Location ** Primary paper (page, table, appendix)	† Other (paper or protocol, website (URL)
**WHAT: materials**	1	Detailed description of the type of exercise equipment (e.g. weights, exercise equipment such as machines, treadmill, bicycle ergometer etc)		Fraga et al., 2021.doi:104103/1673-5374.313067
**WHO: provider**	2	Detailed description of the qualifications, teaching/supervising expertise, and/or training undertaken by the exercise instructor	Page 7	
**HOW: delivery**	3	Describe whether exercises are performed individually or in a group	Page 7	
	4	Describe whether exercises are supervised or unsupervised and how they are delivered	Page 7	
	5	Detailed description of how adherence to exercise is measured and reported	Page 7	
	6	Detailed description of motivation strategies	Page 11	
	7a	Detailed description of the decision rule(s) for determining exercise progression	Page 7	
	7b	Detailed description of how the exercise program was progressed	Page 7	
	8	Detailed description of each exercise to enable replication (e.g. photographs, illustrations , video etc)		Marmeleira et al., 2018. doi:10.1016/ j.gerinurse.2017.10.015
	9	Detailed description of any home program component (e.g. other exercises, stretching etc)	N/A	
	10	Describe whether there are any non-exercise components (e.g. education, cognitive behavioural therapy, massage etc)	Page 7	Fraga et al., 2021. doi: 104103/1673-5374.313067
	11	Describe the type and number of adverse events that occurred during exercise	N/A	

**WHERE: location**	12	Describe the setting in which the exercises are performed	Page 7	
**WHEN, HOW MUCH: dosage**	13	Detailed description of the exercise intervention including, but not limited to, number of exercise repetitions/sets/sessions, session duration, intervention/program duration etc	Page 7	Fraga et al., 2021. doi: 10410 3/1673-5374.31 3067
**TAILORING: what, how**	14a	Describe whether the exercises are generic (one size fits all) or tailored whether tailored to the individual	Page 7	
	14b	Detailed description of how exercises are tailored to the individual	Page 7	
	15	Describe the decision rule for determining the starting level at which people commence an exercise program (such as beginner, intermediate, advanced etc)		Marmeleira et al., 2018. doi:10.1016/ j.gerinurse.2017.10.015
**HOW WELL: planned, actual**	16a	Describe how adherence or fidelity to the exercise intervention is assessed/measured		Marmeleira et al., 2018. doi:10.1016/ j.gerinurse.2017.10.015
	16b	Describe the extent to which the intervention was delivered as planned	Pages 7 and 11	

***It is recommended that this checklist is used in conjunction with the Explanation and Elaboration Statement which is a guide each item in the CERT Checklist**

The CERT Checklist is designed for reporting details of an exercise intervention. The CERT Checklist should be used in conjunction with a reporting checklist appropriate for the study type e.g. the CONSORT Statement (www.consort-statement.org) for randomised controlled trials, the SPIRIT Statement (www.spirit-statement.org) for a clinical trial protocol. For further guidance regarding reporting guidelines please consult the EQUATOR network (www.equator-network.org)

** Authors – please use N/A if an item is not applicable   Reviewers – please use "?" if information is not provided or not/insufficiently reported

† If the information is not provided in the primary paper that is under consideration, please provide details of where this information is available e.g. in a published protocol, published papers (provide citation details) or on a website (provide the URL).

Marmeleira et al., 2018. [illegible]
